# ASCT2 overexpression is associated with poor survival of OSCC patients and ASCT2 knockdown inhibited growth of glutamine‐addicted OSCC cells

**DOI:** 10.1002/cam4.2965

**Published:** 2020-03-12

**Authors:** Yijun Luo, Wei Li, Zihang Ling, Qinchao Hu, Zhen Fan, Bin Cheng, Xiaoan Tao

**Affiliations:** ^1^ Guangdong Provincial Key Laboratory of Stomatology Department of Oral Medicine Guanghua School of Stomatology Sun Yat‐sen University Guangzhou P.R. China; ^2^ Department of Experimental Therapeutics The University of Texas MD Anderson Cancer Center Houston TX USA

**Keywords:** ASCT2, glutamine, oral squamous cells carcinoma, ROS

## Abstract

**Background:**

Alanine‐serine‐cysteine transporter 2 (ASCT2), a major glutamine transporter, is essential for cell growth and tumor development in a variety of cancers. However, the clinicopathological significance and pathological role of ASCT2 in OSCC (oral squamous cell carcinoma) lesions remain unclear.

**Methods:**

Sections from 89 OSCC patients and 10 paracancerous tissue controls were stained by immunohistochemistry (IHC) to detect the expression of ASCT2, glutaminase, and Ki‐67. Survival analysis was carried out to determine the predictive value of ASCT2 expression using the log‐rank test. Moreover, the critical role of ASCT2 in tumor growth was determined by a series of in *vitro* and in *vivo* assays. Cell Counting Kit‐8 (CCK8), Western Blotting (WB), Reactive Oxygen Species (ROS), and Glutathione (GSH) detection were applied to explore the molecular mechanism of ASCT2 involvement in tumor development.

**Results:**

In OSCC lesions, ASCT2 expression was significantly increased and associated with cell proliferation index (Ki‐67) and GLS expression. Moreover, survival analysis showed that OSCC patients with high ASCT2 expression had lower overall survival (*P* = 0.0365). In OSCC cell lines, the high level of ASCT2 was inherent and related to the glutamine addiction of tumor cells. In *vitro* and in *vivo* functional experiments revealed that targeted silencing of ASCT2 can effectively inhibit OSCC cell proliferation and tumor growth. Mechanistically, targeting ASCT2 knockdown reduced glutamine uptake and intracellular GSH levels, which contribute to the accumulation of ROS and induce apoptosis in OSCC cells.

**Conclusion:**

ASCT2 is a significant factor for predicting overall survival in patients with OSCC, and targeting ASCT2 to inhibit glutamine metabolism may be a promising strategy for OSCC treatment.

## Graphical abstract

This study identifies ASCT2 is a significant prognostic factor for predicting overall survival of OSCC patients. Targeted ASCT2 knockdown effectively inhibits cancer growth in vitro and in vivo via inhibiting GSH synthesis and inducing ROS‐mediated apoptosis. Targeting ASCT2 may provide a novel strategy for OSCC treatment.

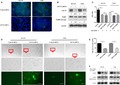

## 1 INTRODUCTION

Glutamine (Gln) is the most abundant amino acid in human plasma. In cancer cells, Glutamine provides a source of both carbon and nitrogen for the synthesis of biological macromolecules. In addition, glutamine produces glutamate due to the action of glutaminase (GLS), which then enters the tricarboxylic acid cycle in the form of alpha‐ketoglutarate to produce ATP, thereby providing energy for the rapid proliferation of tumor cells. Glutamine can also increase the antioxidant defense capacity of cells by participating in the synthesis of glutathione.^1^, ^2^ In addition, it has been shown that glutamine is transported into cells through a surface transporter, promoting essential amino acid influx into the cells via LAT1, activating the p‐mTOR/p‐S6 signaling pathway, and subsequently promoting cell proliferation.^3^, ^4^

Alanine‐serine‐cysteine transporter 2 (ASCT2) is an Na^+^‐dependent neutral amino acid transporter located in the cell membrane. Previous studies have shown that ASCT2 is highly expressed in a variety of tumors such as breast cancer, prostate cancer, melanoma, colorectal cancer, pancreatic cancer, tongue cancer, and lung cancer.^5^, ^6^, ^7^, ^8^, ^9^, ^10^, ^11^ It was reported that ASCT2 mediates the transport of amino acids and plays an important role in glutamine transport in tumor cells.^12^ GLS is a key regulatory enzyme involved in glutamine metabolism that converts glutamine into glutamate.^13^ A previous publication reported that GLS is a mitochondrial amido‐hydrolase enzyme and is considered a ‘gate‐keeper’ enzyme in glutamine metabolism.^14^

Given the critical role of ASCT2 in glutamine transport, some studies have explored the in *vitro* and in *vivo* effects of targeted interference with ASCT2 in a variety of tumor types. Studies have shown that targeting ASCT2‐mediated glutamine uptake via specific inhibitors or ASCT2‐siRNA can reduce tumor growth and development in endometrial, prostate, and colorectal cancer.^7^, ^10^, ^15^ Furthermore, previous studies have reported that ASCT2 may be used as a potential molecular marker to predict poor prognosis in non–small cell lung cancer and esophageal squamous cell carcinoma.^8^ In breast cancer, high expression of ASCT2 is associated with poor recurrence‐free survival.^16^

It is known that head and neck squamous cell carcinoma (HNSCC) is the sixth‐most common malignant tumor worldwide.^17^ As the main type of HNSCC, oral squamous cells carcinoma (OSCC) still lacks effective prognostic indicators and its 5‐year survival has not risen significantly (still <50%).^18^, ^19^, ^20^, ^21^ Therefore, investigators worldwide are seeking novel biomarkers for OSCC treatment to improve survival rates. Recently, studies have demonstrated that ASCT2 and GLS are highly expressed in OSCC lesions compared with normal tissues, which suggests that ASCT2‐mediated glutamine transport is related to the development of OSCC. 7, 19, 22 However, the effect of targeting ASCT2 to treat OSCC tumors due to their dependence on glutamine had yet to be delineated.

Therefore, we conducted this study to investigate the expression of ASCT2 in OSCC in a Chinese population and explore the relationship between ASCT2 expression and clinical features, as well as biological parameters such as GLS and Ki67 (a biomarker used to evaluate cell proliferation).

## 2 MATERIALS AND METHODS

### 2.1 OSCC patients

We analyzed samples from a total of 89 patients with OSCC, including a tissue microarray with 45 samples of OSCC, 8 samples of normal tissues obtained from Alenabio Co. (Cohort 1), and 44 samples of OSCC which were collected from the Hospital of Stomatology, XXXX University between 2003 and 2010 (Cohort 2). This study was approved by the institutional review board. All patients were informed and agreed with the sample collection. For all patients, no chemotherapy or radiotherapy was performed before surgery. The day of surgery was taken as day 1 when accessing postoperative survival. The follow‐up duration ranged from 6 to 148 months (median, 86 months). Table 1 lists the baseline clinicopathological characteristics of Cohort 1 and Cohort 2. The fifth edition of the AJCC Cancer Staging Manual was used to stage the samples.

**TABLE 1:** Baseline clinicopathological characteristics of Cohort 1 and Cohort 2

Clinical parameter	Cohort 1 (n = 45)	Cohort 2 (n = 44）
Age		
≤65 years/＞65 years	37/8	36/8
Gender		
Male/female	29/16	38/6
UICC stage		
I‐II	38	28
III‐IV	7	15
		1
Tumor size		
T1‐T2	40	37
T3‐T4	5	6
		1
N stage (lymph node metastasis)		
N0	41	31
N1‐N2	4	12
		1
Grading		
G1	25	26
G1‐2	2	7
G2	13	2
G3	4	9
Others	1	
Release		
Yes	—	12
No	—	23
Unknown	—	9

### 2.2 Immunohistochemical staining

We used the 3,3'‐diaminobenzidine method (DAB kit) for immunohistochemical analysis of paraffin sections. The ASCT2 and Ki67 monoclonal antibodies were purchased from Cell Signaling Technology, Inc. The GLS monoclonal antibody was purchased from Abcam, Inc. The DAB kit was obtained from GeneTech Company Limited. The immunohistochemical staining was performed according to the manufacturer's instructions. The ASCT2, GLS, and Ki67 expression scores were assessed as reported in a previous study.^5^, ^8^, ^16^

### 2.3 Cell lines

A total of 6 human OSCC cell lines (UMSCC1 (UM1), SCC15, HSC4, CAL33, HSC3, and HSC6) were maintained at 37°C and 5% CO_2_ in DMEM/F12 (1:1) (Gibco, Invitrogen Corp) supplemented with 10% FBS and different concentrations of glutamine. The SCC15 and human normal oral keratinocyte (HOK) cell lines were purchased from ATCC. The HSC3 and HSC4 cell lines were gifts provided by YYYY Professor (XXXX University, China). The UM1 cell line was obtained from YYYY Professor (Oral and Maxillofacial Surgery department, XXXX University, China) and HSC‐6 and CAL33 cells were kindly donated by YYYY Professor (XXXX Institute of Dental and Craniofacial Research, USA).

### 2.4 Cell proliferation assay

CCK8 cell proliferation was analyzed using the Cell Counting Kit‐8 (CCK‐8) as reported in the previous study.^23^ In brief, cells were seeded into 96‐well plates, CCK8 (10 µL) mixed with 100‐µL medium was added into each well, and plates were incubated at 37°C for 1 hour. Then, the absorbance was measured at 450 nm using an automated microplate reader (Thermo Fisher Scientific). All experiments were conducted in triplicate.

### 2.5 Wound healing assay

UM1 and SCC15 cells were seeded in 6‐well plates (7 × 105 cells/well) and cultured overnight. When a monolayer of cells had spread over the bottom of the well, a 200‐micron tube was used to vertically scratch the cell layer. After washing the cell suspension and cell fragments with PBS, the cells were cultured in serum‐free medium to allow the wound to heal. Phase contrast images of the same position were taken under an inverted microscope at 0 and 24 hours. Three repeated experiments were performed.

### 2.6 Western blotting

Western blotting was performed as previously described to detect protein expression.^24^ The cells were harvested and lysed using RIPA buffer (sigma‐Aldrich) with 1% protease inhibitors. The same amount of protein was loaded onto a gel for separation and then transferred to a PVDF membrane. The ECL substrate (Millipore) was used to visualize the signal. All primary antibodies were diluted 1:1000, and the secondary antibody 1:3000. The primary antibodies were purchased from Cell Signaling Technology, including the rabbit monoclonal antibody ASCT2 (D7C12), PARP (46D11), and β‐Actin (13E5).

### 2.7 siRNA duplexes and transfection

siRNA oligonucleotide duplexes for ASCT2 were purchased from sigma‐Aldrich Co. The targeting sequences were as follows: siRNA#1 (CCGGGCTGCTTATCCGCTTC‐ TTCAACTCGAGTTGAAGAAGCGGATAAGCAGCTTTTT), siRNA#2 (CCGGC‐ TGATTATGAGGAATGGATACTCGAGTATCCATTCCTCATAATCCAGTTTTTG). The siRNA oligonucleotides were transfected into the target cells with Lipofectamine 3000 (Life Technologies) according to the manufacturer's instructions, as previously described.^6^

### 2.8 Intracellular glutathione assay

Intracellular glutathione was measured using a glutathione assay kit from Cayman Chemical as previously described.^22^ After collection, cells were resuspended in MES buffer, sonicated, and deproteinated by precipitation with an equal volume of 10% metaphosphoric acid. The supernatant was collected via centrifugation at 5000 rpm for 5 minutes, neutralized with 0.2‐M triethanolamine, and then assayed for GSH according to the manufacturer's instructions.

### 2.9 ROS detection

Intracellular ROS were detected using the total ROS detection kit (Enzo Life Sciences) as we previously described.^17^ In brief, cells were seeded in a 12‐well plate. When the cell density reached approximately 60%, the cells were treated and then stained with ROS detection solution at 37°C for 1 hour. Finally, the cells were observed under a fluorescence microscope.

### 2.10 shRNA duplexes and transfection

Recombinant lentiviral transfer vectors: psi‐LVRU6GH encoding shRNA specific for human ASCT2 were designed and prepared by Genecopoeia. The sequences were as follows: forward GATCCGGGATCTTGCGAGAAATATCTTTCAAGAGAAGATA TTTCTCGCAAGATCCTTTTTTTGGAATT, reverse: AATTCCAAAAAAGGATC TTGCGAGAAATATCTTCTCTTGAGGATCTTGCGAGAAATATCTTCGGATC. SCC15 cells were transfected with the lentiviruses according to the manufacturer's instructions. Hygromycin (500 µg/mL) was used to screen for stably expressing cell lines 48 hours later. Quantitative real‐time PCR and WB were used to detect the expression of ASCT2. Total RNA was extracted with Trizol (Invitrogen) for reverse transcription according to the manufacturer's protocol. Reverse transcription reaction for cDNA synthesis was performed using the PrimeScript^TM^ RT Master Mix (Takara, Dalian, China). The measurement of cDNA was conducted using the SYBR green kit as directed by the manufacturer (Roche). The calculation of relative levels was done according to the 2^−ΔΔCt^ method. Protein levels were normalized to GAPDH. The primers of ASCT2 were as follows: forward: TAAACCCACATCCTCCATCT, reverse: GCTG CTTATCCGCTTCTTC.

### 2.11 eGFP/Fluc cell transduction

A recombinant lentiviral vector encoding luciferase was designed by Inovogen Tech. Co. The shControl and shASCT2 SCC15 cells were incubated with the luciferase lentivirus for 12 hours before the media were replaced with fresh media. Luciferase expression was detected by WB and luciferase substrate (Luciferin). Puromycin (2.5 µg/mL) was used to screen for stably expressing cell lines 48 hours later.

### 2.12 Xenograft mouse models

The 6‐week‐old BALB/c female nude mice, purchased from XXXX University Laboratory Animal Center, were randomly divided into 2 groups: shControl and shASCT2 (n = 10/group). The SCC15‐luc shControl or shASCT2 cells were injected subcutaneously into the right flanks of the mice. A luciferase signal in the shControl and shASCT2 tumors was detected after 2 and 36 days of transplantation, respectively. Subcutaneous tumor growth was monitored every 3 days, and the mice were sacrificed at 36 days.

### 2.13 Statistical analysis

Statistical analyses were performed using IBM SPSS Statistics 20. A *P* < 0.05 was applied to indicate a statistically significant difference. Comparisons between different groups were performed using student's *t* test or one‐way ANOVA as appropriate, unless otherwise specified. The comparison between groups was evaluated by *X* ^2^ test and Fisher's exact test. The correlation between the indicators was analyzed using the nonparametric spearman rank test.

The clinical stages of disease were divided into 2 groups: stage I or II (early stage) and stage III or IV (advanced stage). Survival rates were assessed by the Kaplan‐Meier method and survival differences were analyzed by log‐rank test. Overall survival (OS) was defined as the time interval from the day of surgery to death. Progression‐free survival (PFS) was defined as the time from the day of surgery to the first relapse.

## 3 RESULTS

### 3.1 ASCT2 expression was significantly upregulated and associated with overall survival in OSCC patients

**FIGURE 1:**
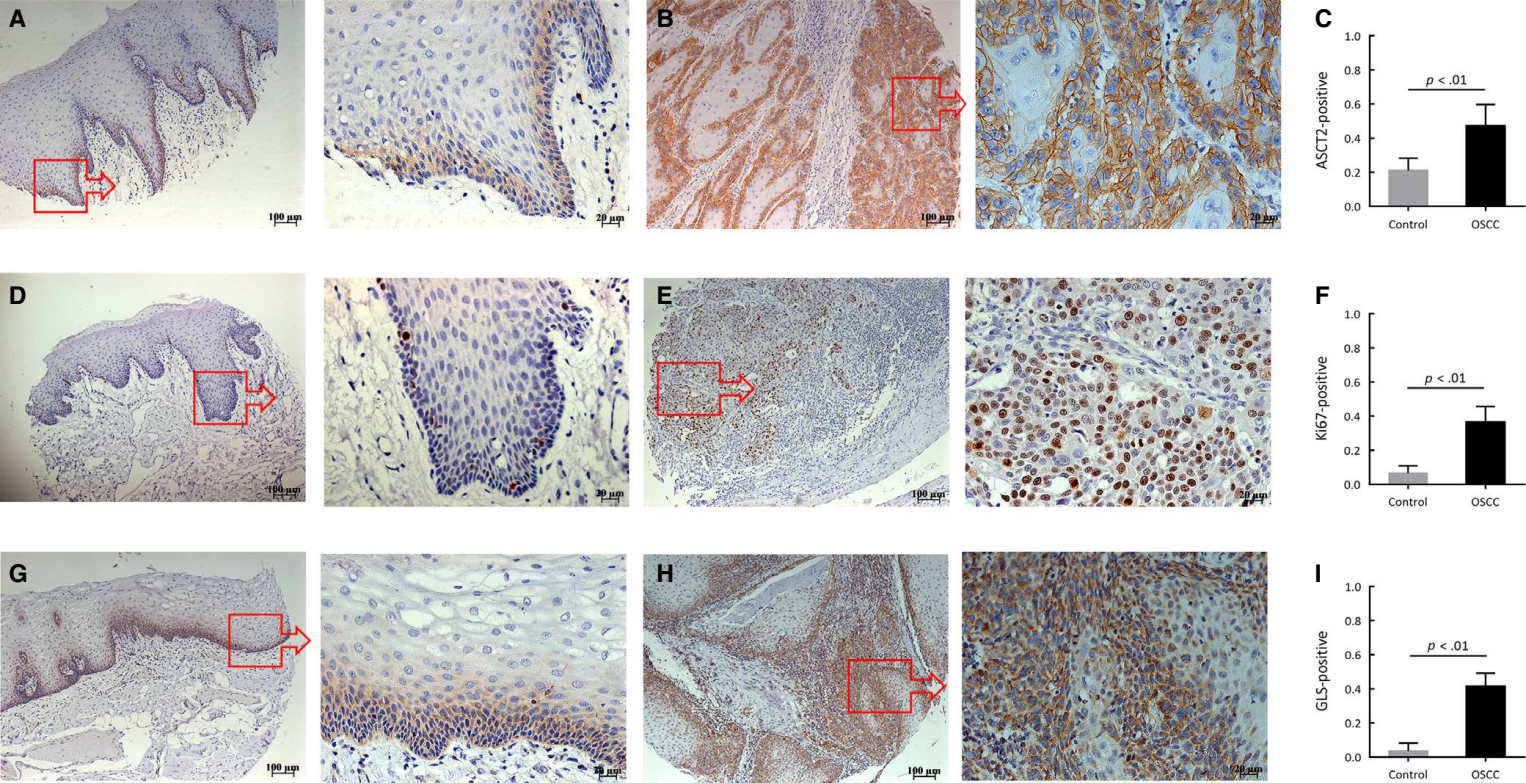
Representative examples of ASCT2, Ki67, and GLS staining in oral squamous cell carcinoma (OSCC) and normal tissues. The figure shows the relative expression of ASCT2 (A and B), Ki67 (D and E), and GLS (G and H) between normal tissues and OSCC lesions. As figures (C, F, and I) show, the expression of ASCT2, Ki‐67, and GLS in OSCC lesions was significantly higher than in normal controls (*P* < 0.01). The square box demonstrates the area of interest (×100‐fold, right panel), which is also shown at a larger magnification (×400‐fold, left panel). ASCT2, alanine‐serine‐cysteine transporter 2; GLS, glutaminase

**FIGURE 2:**
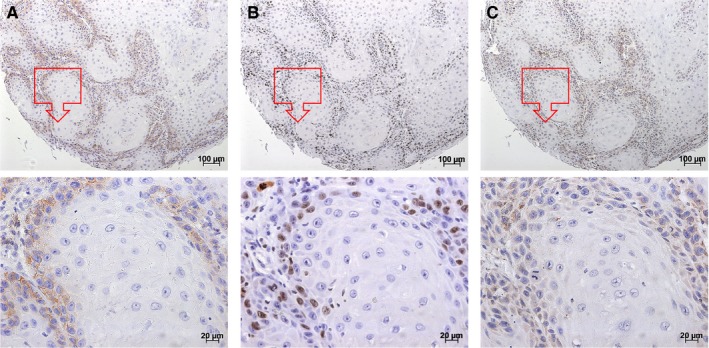
Representative examples of ASCT2, Ki67, and GLS staining in the same OSCC sample. The figure shows that the staining position in ASCT2 (A), Ki67 (B), and GLS (C) was similar in OSCC lesions. These three biomarkers were mainly expressed in the periphery of the tumor bud. The square box demonstrates the area of interest (×100‐fold, the panel above), which is also shown at a larger magnification (×400‐fold, the panel below). ASCT2, alanine‐serine‐cysteine transporter 2; GLS, glutaminase; OSCC, oral squamous cell carcinoma

To determine whether glutamine is metabolized differently compared with paracancerous normal controls, glutamine metabolism‐related proteins including ASCT2, GLS, and Ki‐67 were measured in OSCC specimens. First, we performed immunohistochemical staining on a human OSCC tissue microarray. As is shown in Figure 1, the ASCT2 protein was mainly detected in the membrane of the OSCC epithelial cells. At all stages of OSCC cancer, ASCT2, GLS, and Ki‐67 protein levels were significantly increased compared with normal controls (Figure 1). Moreover, Spearman correlation analysis showed that positive expression of ASCT2 was significantly associated with GLS level (*P* = 0.0389) and Ki‐67 LI (*P* = 0.008). Interestingly, we found that the staining position of these three biomarkers was similar and mainly localized in the periphery of the tumor bud. Figure 2 shows representative examples of immunohistochemical staining of ASCT2, GLS, and Ki‐67 in the same OSCC sample.

**FIGURE 3:**
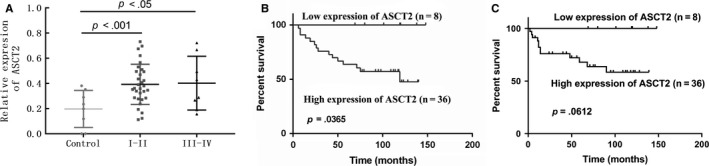
The comparison between ASCT2 expression and the pathological stage of OSCC patients and Kaplan‐Meier survival curve of patients with low and high ASCT2 expression. (A) Comparison of ASCT2 protein levels between normal controls and different stages of OSCC lesions. The result shows ASCT2 level was significantly associated with tumor stage. (B) A statistically significant difference in OS was observed between the patients with low and high ASCT2 tumor expression (*P* = 0.0365). (C) No statistically significant difference in PFS was observed between the patients with low and high ASCT2 tumor expression (*P* = 0.0612). ASCT2 alanine‐serine‐cysteine transporter 2; OSCC, oral squamous cell carcinoma

In the 44 samples from cohort 2, the 5‐year survival rate was 67.5% and median survival time exceeded 6.5 years in all patients. The results of univariate and multivariate analyses are shown in Table 2. Overall patient survival was significantly associated with age, relapse, and pathological stage as assessed by univariate analysis. Multivariate analysis confirmed that age and pathological stage were independent prognostic factors for poor OS. As Figure 3A shows, ASCT2 level was significantly associated with tumor stage. In addition, Figure 3B and 3C show the Kaplan‐Meier survival curve of patients with low and high ASCT2 expression. A statistically significant difference in OS was observed between the patients with low and high ASCT2 tumor expression (*P* = 0.0365). No statistically significant difference in PFS was observed between the patients with positive ASCT2 and those with negative ASCT2 tumor expression (*P* = 0.0612).

**TABLE 2:** Univariate and multivariate analysis of overall survival in oral squamous cell carcinoma (OSCC) patients

Overall survival			
Variables	Univariate analysis		Multivariate analysis
	5‐year survival rate (%)	HR 95% CI *P*‐value	HR 95% CI *P*‐value
Age		3.124	3.930
≤65 years	75	1.033‐9.449	1.241‐12.446
＞65 years	37	**0.044**	**0.020**
Gender		1.075	
Male	70	0.240‐4.809	
Female	50	0.924	
Relapse		1.137	
Yes	54	1.005‐1.287	
No	80	**0.042**	
Pathological stage		5.889	6.430
Ⅰ or Ⅱ	79	1.821‐19.044	1.977‐20.909
Ⅲ or Ⅳ	46	**0.003**	**0.002**
ASCT2		30.692	
High	60	0.164‐5756.427	
Low	100	0.200	

Bold values indicate the statistically significant factors in the survival analysis.

These human specimen data suggest that a higher ASCT2 level may play an important role in tumor development and may be a potential prognostic biomarker in OSCC patients.

### 3.2 ASCT2 expression was significantly increased and associated with glutamine addiction in OSCC cells

**FIGURE 4:**
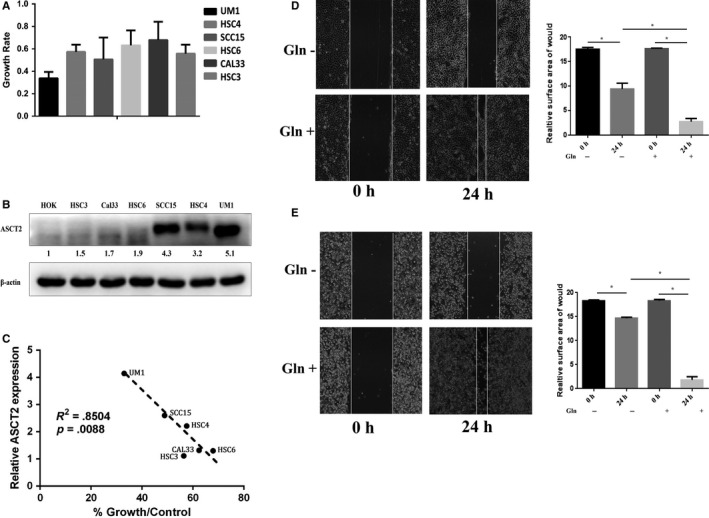
ASCT2 expression was positively correlated with glutamine addiction in OSCC. (A) CCK8 assays were conducted to determine the growth of 6 OSCC cell lines (UM1, HSC4, SCC15, HSC6, CAL33, and HSC3) cultured in a glutamine‐free environment compared with HOK cell line. (B) Higher ASCT2 expression in the 6 OSCC cell lines compared with the HOK cell line was determined by Western blotting. (C) Spearman correlation demonstrated that cell growth in a glutamine‐free environment was negatively associated with ASCT2 expression (*P* = .0088). The result indicates that ASCT2 expression was significantly associated with the addiction of glutamine in OSCC cells. In addition, wound healing assays were used to confirm the glutamine addiction of UM1 (D) and SCC15 cells (E) (*P < 0.01). ASCT2, alanine‐serine‐cysteine transporter 2; Gln, glutamine; OSCC, oral squamous cell carcinoma

Many types of cancer cells exhibit addiction to glutamine during their growth and metastasis.^1^, ^2^, ^3^, ^4^, ^5^, ^6^, ^7^, ^8^ To confirm this characteristic of OSCC cells, 6 OSCC cell lines were cultured under different concentrations of glutamine in vitro. The CCK8 results showed that the proliferation of OSCC cells was significantly inhibited under glutamine deprivation (Figure 4A). To confirm the glutamine addiction of OSCC cells, wound healing assays were conducted in the SCC15 and UM1 cell lines. The results demonstrated that deprivation of glutamine effectively inhibited the proliferation and migration of OSCC cells (Figure 4B). All these data suggested that OSCC cells are addicted to glutamine.

As previous studies reported, ASCT2 is the main transporter of glutamine in several cancer cell types. Although our study revealed increased ASCT2 expression in OSCC lesions, the inherent ASCT2 expression of OSCC cancer cells had yet to be determined in vitro. To verify inherent ASCT2 expression, an increased ASCT2 level in several OSCC cell lines was confirmed when compared with a normal epithelial cell line. Simultaneously, we observed that ASCT2 expression is clearly different between the 6 OSCC cell lines (Figure 4C). Moreover, we further found that deprivation of glutamine inhibited growth in a time‐dependent manner in ASCT2 high‐expressing cells while ASCT2 low‐expressing cells demonstrated less dependence on glutamine (Figure 4D, E). Spearman correlation analysis showed that ASCT2 expression was significantly associated with the addiction of glutamine in OSCC cells (*P* = 0.0088). These findings suggest a positive correlation between the addiction of glutamine and the level of ASCT2 in OSCC cells.

### 3.3 Targeted ASCT2 knockdown inhibited GSH synthesis and induced apoptosis via accumulation of intracellular ROS

**FIGURE 5:**
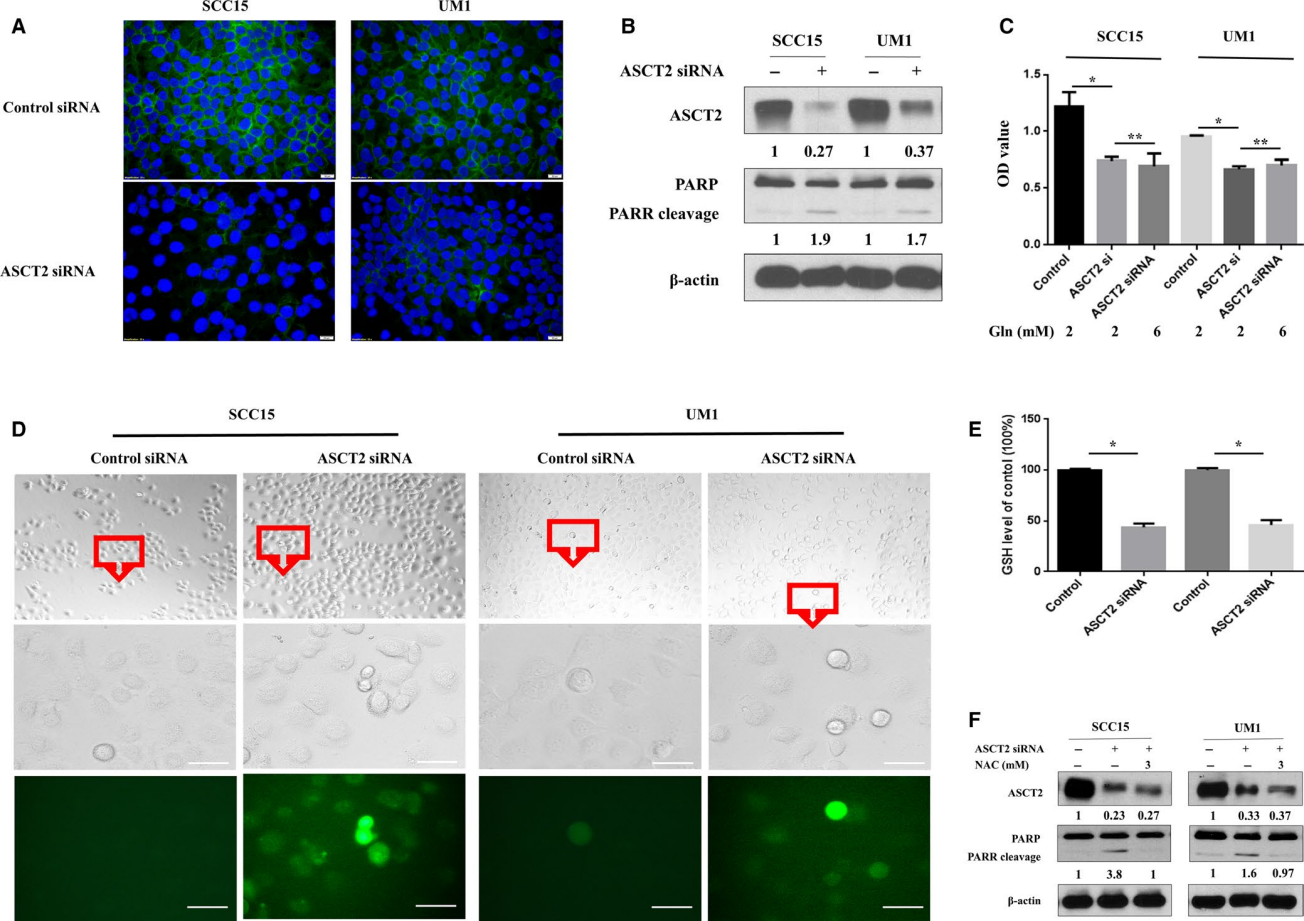
ASCT2 knockdown induced apoptosis by suppressing GSH synthesis, thus increasing ROS levels in OSCC. Immunofluorescence staining (A) and Western blotting (B) were used to determine the downregulation of ASCT2 in SCC15 and UM1 cells transfected with ASCT2 siRNA. Moreover, the decrease in ASCT2 was accompanied by increased apoptosis in ASCT2‐knockdown cells (B). CCK8 assays (C) were performed to determine cell growth in SCC15 and UM1 cells under different glutamine concentrations. The proliferation of ASCT2‐knockdown OSCC cells was significantly inhibited compared with the nontargeted siRNA controls and cannot be reversed by adding extra glutamine (6 mmol/L) to the culture medium. Intracellular ROS were stained with a commercial ROS detection kit, and higher ROS levels were observed in OSCC cells transfected with ASCT2 siRNA (D, bar: 50 µm). The intracellular levels of GSH were significantly decreased in SCC15 and UM1 cells transfected with ASCT2 siRNA compared with the control groups (E). NAC, a ROS inhibitor, effectively inhibited apoptosis ROS induced in ASCT2‐knockdown cells (F). *P < 0.05; NS: no statistical significance. ASCT2, alanine‐serine‐cysteine transporter 2; Gln, glutamine; GSH, glutathione; NAC, N‐acetylcysteine; PARP, poly‐ADP‐ribose polymerase

To determine the potential role of ASCT2, we used two different siRNAs to decrease ASCT2 expression in SCC15 and UM1 cells. A significant decrease in ASCT2 levels was observed in SCC15 and UM1 cells transfected with ASCT2‐specific siRNAs compared with a nontargeted siRNA control (Figure 5A, B). Then, CCK8 assay was conducted in ASCT2‐knockdown SCC15 and UM1 cells to determine whether ASCT2 function is critical to the proliferation OSCC cells in vitro (Figure 5C). Results showed that the proliferation of ASCT2‐knockdown OSCC cells was significantly inhibited compared with the nontargeted siRNA controls. This was like the effect of glutamine deprivation on OSCC cells. In addition, we also observed that inhibition of cell proliferation cannot be reversed by adding extra glutamine to the culture medium in ASCT2‐knockdown OSCC cells (Figure 5C).

Previous studies have reported that the majority of intracellular glutamine is converted into glutamate, which can be utilized for glutathione production and protection against oxidative stress.^25^, ^26^, ^27^, ^28^ We, therefore, set out to determine whether ASCT2‐knockdown changes apoptosis due to the decrease in glutamine uptake and GSH, which results in intracellular ROS accumulation. As shown in Figure 5A,B, ASCT2 expression was clearly downregulated after targeted knockdown of ASCT2 in OSCC cells. As expected, the decrease in ASCT2 was accompanied by a reduced intracellular uptake of glutamine and a reduced intracellular level of glutathione in ASCT2‐knockdown cells compared with control OSCC cells (Figure 5E). These results confirmed that ASCT2 is a main transporter of glutamine of OSCC cells. Moreover, an increased ROS level was detected in ASCT2‐knockdown cells (Figure 5D). The percent of ROS‐positive cells in OSCC with ASCT2 siRNA was significantly upregulated compared with Controls (SCC15: 59.80 ± 9.64 vs. 2.33 ± 0.66, *P* = 0.004; UM1: 67.03 ± 6.35 vs. 4.5383 ± 0.58, *P* = 0.006). Next, we explored whether ASCT2 knockdown–induced apoptosis in OSCC cells was due to the upregulation of intracellular ROS levels. As shown in Figure 5F, NAC (sigma‐Aldrich), a precursor of glutathione, largely abolished the apoptosis of ASCT2‐knockdown cells, as indicated by the appearance of PARP cleavage. Based on the findings shown in Figure 5, we concluded that ASCT2‐knockdown–induced apoptosis by upregulating intracellular ROS levels due to decreased glutamine uptake and glutathione levels in OSCC cells.

### 3.4 Targeted ASCT2 knockdown inhibited tumor growth in OSCC xenografts

**FIGURE 6:**
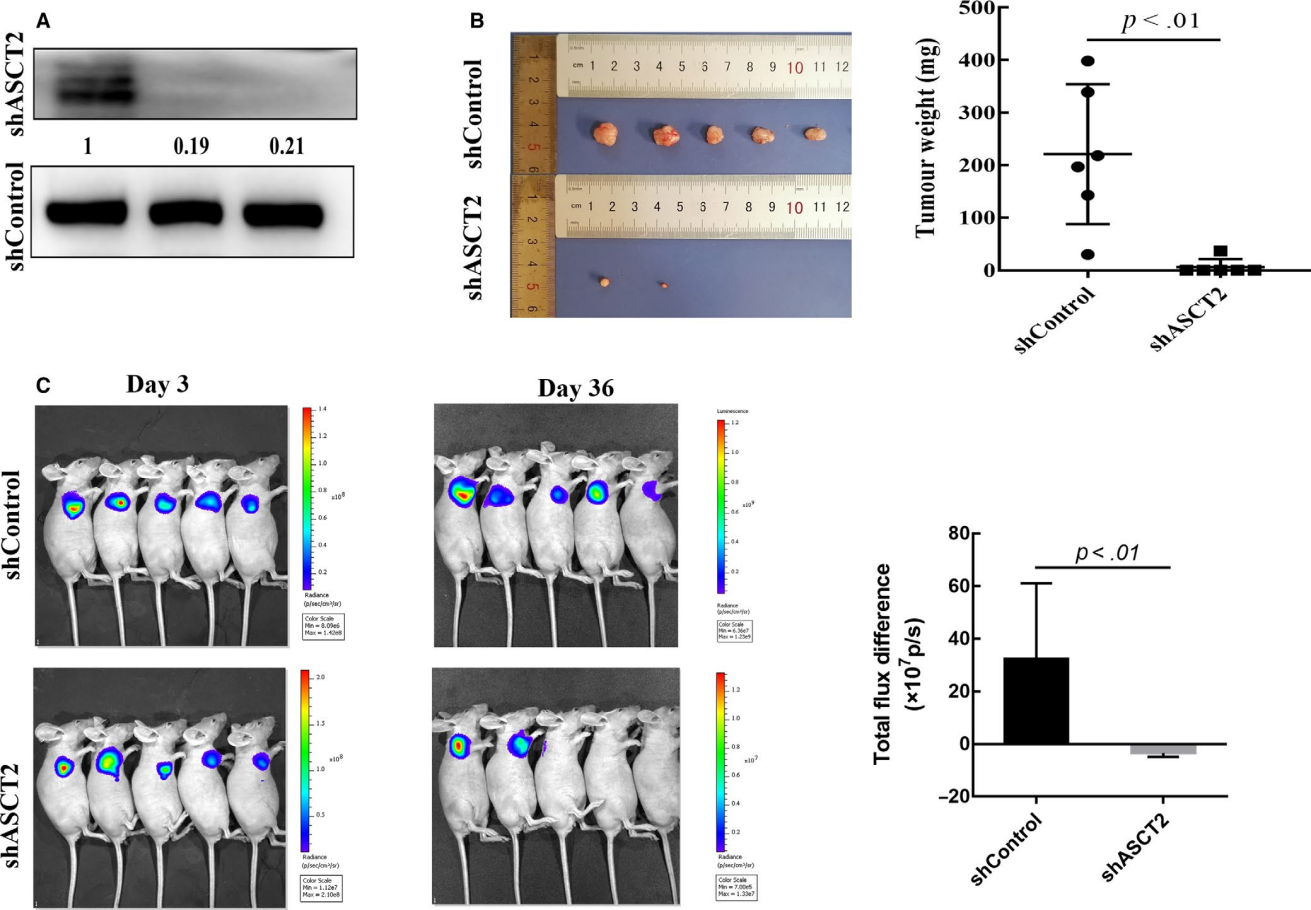
ASCT2 knockdown inhibited tumor growth in vivo. (A) The downregulation of ASCT2 expression in shASCT2 SCC15‐luc cells was confirmed by Western blotting. (B) All mice were euthanized at day 36 and tumors were collected for size and weight measurement. The shASCT2 tumors were found to be significantly smaller than shControl tumors (*P *< 0.01). (C) SCC15‐luc cells stably expressing shControl or shASCT2 were injected subcutaneously into male nude mice. Bioluminescent images were taken on day 3 and day 36. Similar luciferase signals were confirmed in shControl and shASCT2 tumors on day 3. Results of the bioluminescence analysis showed a significant difference between shControl and shASCT2 tumor by day 36 (*P *< 0.01). The significance of the bioluminescent difference was assessed via independent‐sample *t* test (data are Mean ± SD). ASCT2, alanine‐serine‐cysteine transporter 2

To determine whether ASCT2 plays a critical role in tumor growth in vivo, SCC15 cells expressing shControl or shASCT2 were transduced with a lentiviral vector co‐expressing eGFP and firefly luciferase. SCC15‐luc cells were enriched to high purity, resulting in similar GFP/luciferase expression in each shControl and shASCT2 cell line. ASCT2 expression was detected by WB to confirm the downregulation of ASCT2 in shASCT2 cells (Figure 6A). Then, SCC15‐luc cells were subcutaneously injected into nude mice, and similar luciferase signals were confirmed in shControl and shASCT2 tumors 72 hours after transplantation (Figure 6B). Results of the bioluminescence analysis showed a significant decrease in shASCT2 tumor size by day 36 (Figure 6A,B).

Mice were euthanized by carbon dioxide asphyxiation according to the approved ethical protocol after 36 days, due to the size of the shControl tumors. The tumors were photographed and weighed after isolation, and the shASCT2 tumors were found to be significantly smaller than shControl tumors (Figure 6C). Because only two mice in the shASCT2 group had distinguishable xenografts, IHC staining of these xenograft tumors for ASCT2, GLS, and Ki‐67 was not compared with shControl tumors. Together, these in *vivo* experiments suggest that ASCT2 is an effective target for OSCC treatment.

## 4 DISCUSSION

We not only explore the clinical prognostic value of ASCT2 expression in OSCC patients but also the potential of ASCT2 for the treatment of OSCC and its possible molecular mechanisms in vitro and in vivo for the first time. Our research mainly found significant differences in the OS of OSCC patients when comparing patients with low and high expression of ASCT2. Targeted ASCT2 knockdown effectively decreased glutamine uptake and GSH levels, resulting in intracellular ROS accumulation which induced apoptosis in OSCC cells. Moreover, the critical role of ASCT2 in tumor growth was verified in OSCC xenografts. Therefore, ASCT2 may play a critical role in tumor growth by mediating glutamine metabolism, and may serve as a significant prognostic factor in patients with OSCC.

Glutamine plays an important role in cell proliferation, and its metabolic reprogramming often occurs in cancer cells, which manifests as increased glutamine metabolism.^29^ A previous article reported the prognostic significance of amino acid transporter expression (LAT1, ASCT2, and xCT) in surgically resected tongue cancer.^7^ In comparison, our study had the advantage of determining the expression of glutamine metabolic pathway‐related factors in OSCC. This primary study demonstrated that the expression of ASCT2 and GLS was significantly increased in OSCC compared with paracancerous normal mucosa. Moreover, there was a positive correlation between ASCT2 and GLS. All these findings suggested that the increase in glutamine metabolism mediated by ASCT2 and GLS might play an important role in the proliferation of cancer cells in OSCC lesions.

A previous study reported that Ki‐67 is a potential biomarker in OSCC.^29^ This study found that the expression of ASCT2 was significantly related to Ki‐67 LI in OSCC. Furthermore, the expression distribution characteristics of ASCT2, GLS, and Ki‐67 were consistent in most of the OSCC cases, as demonstrated by the fact that all three biomarkers were highly expressed in the marginal bud region of the tumor. These findings are the first to provide morphological evidence that ASCT2‐mediated glutamine metabolism promotes cancer progression by participating in tumor cell proliferation. The existing evidence suggests that ASCT2 can be used as a molecular marker for clinical prognosis in OSCC. Further study should be done to determine whether ASCT2 upregulation in OSCC patients is induced by the tumor microenvironment or is inherent.

In this study, we examined ASCT2 expression and addiction to glutamine in 6 OSCC cell lines. Our results demonstrated that ASCT2 expression in OSCC cells was higher than in HOK cells without stimulation in vitro. This indicated that high expression of ASCT2 in OSCC cells is inherent rather than induced by the tumor microenvironment in OSCC lesions. Moreover, we also found a positive correlation between ASCT2 levels and addiction to glutamine. This similarly supports the idea that high expression of ASCT2 is an intrinsic requirement for increased glutamine metabolism in OSCC cells.

Previous studies reported that ASCT2 is a main glutamine transporter and plays an important role in a variety of tumors.^1^, ^2^, ^3^, ^4^, ^5^, ^6^, ^7^, ^8^ To further clarify the function of ASCT2 in OSCC, we knocked down ASCT2 expression in OSCC cells and evaluated the role of ASCT2 in tumor cell growth in vitro and in vivo*.* We found that targeted ASCT2 knockdown led to downregulation of intracellular GSH levels due to decreased glutamine uptake in OSCC cells. Furthermore, ASCT2 knockdown resulted in an imbalance in redox status and ROS accumulation, which was mainly mediated by decreased GSH content. In addition, we observed that ROS accumulation promoted apoptosis in ASCT2‐knockdown cells. The critical role of ROS was confirmed by the finding that NAC can effectively decrease apoptosis induced via targeted ASCT2 knockdown. More importantly, the pathological role of ASCT2 in OSCC was also confirmed in vivo*,* as targeted ASCT2 knockdown significantly inhibited tumor growth in nude mice. All these findings indicated that ASCT2 has obvious antitumor potential and can be used as a new target in OSCC. If the upregulation of cell proliferation or drug resistance of OSCC overexpressed ASCT2 can be observed in subsequent studies, the potential and prognostic value of ASCT2 in OSCC treatment would be better evaluated.

## 5 CONCLUSIONS

Alanine‐serine‐cysteine transporter 2 can serve as a significant prognostic factor for predicting overall survival in patients with OSCC and may play an important role in the development of OSCC via ASCT2‐mediated glutamine metabolism.

## DISCLOSURES

Yijun Luo, Wei Li, Zihang Ling, qinchao Hu, Zhen Fan, Bin Cheng, and Xiaoan Tao declare that they have no competing interests.

## AUTHORS' CONTRIBUTIONS

Study design: Xiaoan Tao, Bin Cheng; Data acquisition: Yijun Luo, Zihang Ling, Wei Li; Quality control of data and algorithms: Zihang Ling, Wei Li; Data analysis and interpretation: Xiaoan Tao, Bin Cheng; Statistical analysis: Qinchao Hu, Wei Li; Manuscript preparation: Wei Li, Xiaoan Tao, Bin Cheng; Manuscript editing: Xiaoan Tao, Zhen Fan, Bin Cheng; Manuscript review: Xiaoan Tao, Zhen Fan, Bin Cheng.

## Data Availability

Available under request.
